# Effect of Lidocaine Volume and Concentration on Preventing Incidence and Severity of Propofol Injection Pain

**DOI:** 10.5812/ircmj.16099

**Published:** 2014-03-05

**Authors:** Mohammad Gharavi, Alireza Sabzevari, Ehsanolah Ghorbanian, Rasoul Sajadi, Mohsen Akhondi

**Affiliations:** 1 Department of Anesthesiology, Faculty of Medicine, Mashhad University of Medical Sciences, Mashhad, IR Iran; 2 Cardiac Anesthesia Research Center, Imam Reza Hospital, Faculty of Medicine, Mashhad University of Medical Sciences, Mashhad, IR Iran

**Keywords:** Propofol, Lidocaine, Injections, Pain

## Abstract

**Background:**

Propofol is one of common anesthetic drugs used in anesthesia. The most common side effects of propofol are local pain. Pretreatment with lidocaine can reduce propofol injection pain.

**Objectives:**

The aim of the present study was to assess and compare the efficiency of lidocaine 0.4% and 2% in reducing the incidence and severity of propofol injection pain.

**Patients and Methods:**

This was a double blind prospective clinical trial on children 4-8 years old with class ASA I and II candidates who were referred to Dr. Shaikh Hospital in Mashhad for elective surgery. Sample size calculated 50 patients in each groups based on pilot study. 100 children's were randomly divided equally in two groups, who were injected with lidocaine solutions 2% and 0.4% respectively. patient's pain evaluation based on VSD (verbal descriptor scale) and NRS (Numeric Rating Scale) using patient's verbal reaction and behavior namely fretting, hand drag and tearing. The collated data was analyzed.

**Results:**

There was nosignificant difference as to the first three variables (age, gender and weight P > 0.2). The significant difference regarding pain experience in both groups was noteworthy (P > 0.2).

**Conclusions:**

Most of the studies compared lidocaine with other drugs or its efficiency at different doses. Our study is different in that we applied a constant dose of lidocaine in various volumes and concentration. This result shows that lidocaine with the same does but lower concentration and higher volume is more effective in preventing propofol injection pain. Using diluted lidocaine with the dosage of 1 mg/kg and a concentration of 0.4% is an effective way to relieve pain caused by propofol injection in children.

## 1. Background

Propofol is one of the most common anesthetic drugs used for inducting and continuing anesthesia. It is the drug of choice for millions of patients every year because of its rapid onset and short duration of action. Propofol is insoluble in water and is prepared as fat emulsion. The most common side effects of propofol are local pain on injection site as well as blood pressure decline (1). The incidence of propofol pain varies from 28 % to 90% in different studies (2), with a single report of 85% in children (3). The pain can be ranked as severe, an estimate of 5.6 ± 2.3 on VAS pain evaluation system (4).

Different techniques have been employed to reduce propofol injection pain including diluting, heating and cooling the solution as well as pretreatment with a variety of anesthetics namely lidocaine (5), metoclopramide (6), opiates like meperidine (7), tramadol (2), sodium thiopentone (4), nafamostatmesilate (8), and ketamine at varying dosages (9) at varying dosages. Lidocaine seems a promising choice in this respect owing to efficacy, efficiency, accessibility and affordability (5). Yet, it has not been methodically, if empirically, investigated as to the optimal volume, concentration and dosage in alleviating propofol injection site pain

## 2. Objectives

In this study lidocaine was used as the main pretreatment drug to reduce propofol injection pain, with the same dose but at different volume and concentration. The aim of the present study was to assess and compare the efficiency of lidocaine 0.4% and 2% in reducing the incidence and severity of propofol injection pain.

## 3. Patients and Methods

### 3.1. Study Design

This study was a double blind prospective clinical trial on children 4-8 years old with class ASA I and II who were referred to Dr. Shaikh Hospital in Mashhad for elective surgery. Dr Shekh Hospital is special Hospital for children. The inclusion criteria were 4-8 years old children, ASA class I and II, and candidates for elective surgery. The exclusion criteria were contraindication to use propofol or lidocaine, patients with thrombophlebitis, analgesics administration 24 hours prior to the operation and severe mental and neurological disease and neuromuscular disease. We included 100 children (acceptable samples from 138 children) in our study, Then the selected subjects were divided randomly (with regular sampling method) into two equal groups A and B including 50 patients in each groups, who were injected with lidocaine solutions 2% and 0.4% respectively. Dose of lidocaine was 1 mg/kg diluted with normal saline. Patients were also monitored for systolic and diastolic blood pressure changes pulse rate and oxymetry and arythmia using non-invasive techniques such ECG and non-invasive BP measurement prior to anesthesia.

All patients received their shots in their left antecubital vein with a tourniquet tied on their upper arm, which was removed 30 seconds following the lidocaine solution injection. We then started to administer propofol 1 mg/kg in 5 – 10 seconds, (Don Kook, Korea). Only 1/4 of the entire drug solution was initially administered and the rest were given after patient's pain evaluation based on VSD (verbal descriptor scale) and NRS (Numeric Rating Scale) using patient's verbal reaction and behavior namely fretting, hand drag and tearing.

### 3.2. Statistical Analysis

The sampling was conducted in two parts; initially, participants were selected with regular sampling method based on primary pilot study dataand calculated sample sizeand then were randomly divided in two groups. The sample size was calculated by comparing means of difference between groups by confidence interval 95% and study power 80%. We included 100 children (acceptable samples from 138 children) in our study. The collated data with regard to pain intensity and occurrence was analyzed using SPSS 16 Quantitative data was described using mean ± standard deviation whereas P < 0.05 was considered significant.

### 3.3. Ethical Consideration

After approval was obtained from the local ethical committee of Mashhad University of Medical Sciences and informed consent was obtained from the parents of patients as local protocol , study was initiated and registered in IRCT(code: IRCT201111208143N1). All of the patients were ensured that their privacy will be kept and their personal information will not disclosed in any circumstance.

### 3.4. Limitations

This is chiefly regarded as a pilot study due to the lack of any similar background research in children.

## 4. Results

The data pertaining to patients' age, gender and weight in both groups were shown in Table 1. As can be seen, given the insignificant difference as to the first three variables (age, gender and weight with P values of 0.423 and 0.214 respectively, the significant difference regarding pain experience in both groups was noteworthy. Table 2 compare the two groups in terms of their reaction and thus experience of pain. 22 patients (44%) with pain in group A receiving 0.4% lidocaine compared to 12 patients (22%) who were given 0.2% solutions. VAS and NRS 1-3 was mild pain, 4 - 6 moderate pain and 7 - 10was sever pain.

**Table 1.** Demographic and Characteristics of Study Patients (n = 100) ^a^

	Group A	Group B	Total	P Value
**Age, y**	5.82 ± 1.35	5.51 ± 1.14	5.65 (4-8)	0.218
**Weight, kg**	20.25 ± 6.43	18.66 ± 6.29	19.46 (11-45)	0.214
**Gender**				0.423
Male	26 (52)	30 (60)	56 (56)	
Female	24 (48)	20 (40)	44 (44)	

^a^ Data are presented as mean ± SD or No. (%)

**Table 2.** Compare the Two Groups in Terms of Their Reaction and Thus Experience of Pain

	Group A	Group B	Total	P value
**Pain severity**				0.003
Mild	36	41	77	
Moderate	6	6	12	
Sever	8	3	11	

## 5. Discussion

Propofol, a drug of choice for the induction of anesthesia, can also induce pain on injection site owing to high-fat solubility, thanks to long-chain triglyceride solvents (10). Pain can be either immediate caused by injection site irritation, or delayed, often 15 seconds following injection, via kalikerin and bradykinin systems, vascular dilation and increased permeability to neural terminals (11). A number of studies were conducted, evaluating suggested techniques in this respect. Haugen tried thiopentone sodium whereas Iwama et al. used nafamostatmesilate, a kalikerin inhibitor reportedly effective yet narrowly available due to its high price (8) Likewise, flurbiprofen was reported to relieve propofol injection site pain completely however there is also a matter of cost. Other drugs investigated in other studies to relive propofol injection pain include metoclopramide, and opiates like meperidine (7), tramadol (2), and ketamine (9).

One of the drugs that have been used widely in different trials is lidocaine. Different studies have been performed on lidocaine dosage and way of administration before propofol. Walker et al. In a study published in 2011, administrated lidocaine with tourniquet before propofol in one group, and mixed with propofol in another group. The results were then compared with a group receiving placebo. This study results indicated that propofol injection pain was significantly less in the group who received lidocaine before propofol (P = 0.016) (12).

Khaled also conducted a study on 200 patients in Jordan. He divided patients into 4 groups: the first receiving 4 cc lidocaine 1%, the second group 4 cc (40 mg) paracetamol, the third a mixtures of lidocaine 2% and 100 mg fentanyl, and the forth 4 cc of normal saline. Propofol was administered 60 seconds after pretreatment in all groups. A pain-free injection of 68%, 54%, 70% and 36% were reported respectively. There was not any significant difference between the first and the third group (P > 0.05), but the difference between the forth (placebo) and the other three groups was significant (P > 0.05). There was also a significant difference between the groups receiving lidocaine and the one who received paracetamol (P < 0.05).In conclusion, Khaled reported that lidocaine can relive the propofol injection pain up to 70% (13).

In another study conducted by Beyaz SG in Turkey in 2011, the effect of regular propofol was compared with propofol llipuro with and without lidocaine. 120 children were divided in 4 groups, the first and the second groups received generic propofol, while the other two were given propofol llipuro. Lidocaine was administrated for patients in the second and forth groups. Beyas did not find any significant difference in pain between the first two groups (P > 0.95), but the difference between the third and the forth group (propofol llipuro with and without lidocaine) was significant (P = 0.001) (14). In Sedat Kaya study in 2007, 100 patients with ASA class I and II were divided into 5 groups. lidocaine was administrated as follows; the first group 10 cc of lidocaine 2% mixed with saline without tourniquet, the second group 10 cc of lidocaine 2% mixed with saline with tourniquet for 15 seconds, the third group lidocaine 2% with tourniquet for 30 seconds, the forth group lidocaine 2% with tourniquet for 60 seconds, and fifth group 10 cc normal saline without tourniquet. 90% of patients in group 5 reported pain. Sadat concluded the administration of lidocaine, with or without tourniquet, can significantly reduce the incidence and severity of propofol injection pain compared to normal saline. He also reported that applying tourniquet for 60 seconds substantially augments the alleviating effect of lidocaine (15).

The four groups comprising 368 females subjects in King SY study (New Zealand, 1992) were given the following treatments:

Group 1: 19 cc of propofol ± 1 cc normal saline
Group 2: 1 cc of lidocaine 0.5% (5 mg)
Group 3: 1 cc of lidocaine 1% (10 mg)
Group 4: 1 cc of lidocaine 2% (20 mg)

A dose-dependent link was found between lidocaine and pain reduction (the last pain was reported in group 4) (5).

Jalota et al. preformed a systemic review on 177 clinical trials including 25260 participants to determine the most effective way for decreasing propofol injection pain. He reported an incidence of 60% for pain on propofol injection in general. He stated that using antecubital vein is the most effective single intervention (a relative risk of 0.14% and a confidence interval of 0.07 - 0.30). Jalota recommended that if the hand vein is chosen as the site of injection, pretreatment with lidocaine in conjunction with venous occlusion, or a combined intervention such as pretreatment with ketamine or lidocaine before injection of a propofol emulsion containing medium and long chain triglycerides is highly recommended (16).

Madenoglu et al. also performed a study in Turkey with the aim of evaluating the efficacy of different doses of lidocaine in the prevention of pain due to propofol injection. 120 patients with ASA class I and II were placed in 4 groups; group 1 received only propofol, group 2 a mixture of propofol and 10 mg lidocaine, group 3, 10 mg of lidocaine 30 seconds before propofol and group 4 lidocaine 1 mg/kg before propofol. They reported that the incidence of pain was significantly lower in groups 2 and 4 compared to groups 1 and 3 (P < 0.05 for all). They concluded that a lower dose of lidocaine be more effective, suggesting to mix it with propofol prior to injection (17). Picard and colleagues also performed a systemic review on 56 studies including 6264 patients with 12 different drugs used for reducing propofol injection pain. He concluded that 0.5 mg/kg intravenous lidocaine injected with a tourniquet on the forearm for 30-120 seconds before propofol injection can effectively reduce the pain up to 60% (18). Most of the above studies compared lidocaine with other drugs or its efficiency at different doses. Our study is different in that we applied a constant dose of lidocaine in various volumes and concentration. Also our groups consisted of children with the same demographic background. A significant disparity can be noted between our two groups regarding pain perception [22 patients in group A versus 12 (24%) in group B].

This result shows that lidocaine with the same does but lower concentration and higher volume is more effective in preventing propofol injection pain. In fact, what needs to be the focus of future studies is the lidocaine optimum concentration and volume as only 1 mg/kg lidocaine was used in this study. Madenoglu in his study suggested 10 mg as the effective does for lidocaine. We also need to bear in mind that concentration changes can also affect optimum dosage. What we also need to improve is that the research must include a larger population of subjects, applying more efficient techniques to elicit response to pain as children are often less capable to do so on questioning. Using diluted lidocaine with the dosage of 1 mg/kg and a concentration of 0.4% is an effective way to relieve pain caused by propofol injection in children. Further investigations seem noteworthy owing to the flaws in this study: limited number of participants, poor response elicitation techniques to pain and unblind investigators. A larger blind randomized trial using more efficient elicitation techniques is recommended.
